# Molecular characterization and expression patterns of a non-mammalian toll-like receptor gene (TLR21) in larvae ontogeny of common carp (*Cyprinus carpio* L.) and upon immune stimulation

**DOI:** 10.1186/s12917-018-1474-4

**Published:** 2018-05-03

**Authors:** Hua Li, Ting Li, Yujie Guo, Yujun Li, Yan Zhang, Na Teng, Fumiao Zhang, Guiwen Yang

**Affiliations:** Shandong Provincial Key Laboratory of Animal Resistance Biology, College of Life Sciences, Shandong Normal University, No. 88 East Wenhua Road, Jinan, 250014 People’s Republic of China

**Keywords:** Common carp (*Cyprinus carpio* L.), TLR21, Evolutionary relationship, Expression pattern, Innate immunity

## Abstract

**Background:**

In the host innate immune system, various pattern recognition receptors (PRRs) recognize conserved pathogen-associated molecular patterns (PAMPs) and represent an efficient first line of defense against invading pathogens. Toll-like receptors (TLRs) are a major class of PRRs, which are able to recognize a wide range of PAMPs and play a central role in initiating innate immune responses. TLR21 is one of the non-mammalian TLRs identified in some bird and fish species.

**Results:**

In the present study, we reported the cloning and identification of a TLR21 cDNA from the head kidney of common carp (*Cyprinus carpio* L.), named CcTLR21. The full-length CcTLR21 cDNA was 3557 bp long, including an open reading frame (ORF) of 2895 bp, which encoded a putative protein of 964 amino acids. The putative CcTLR21 protein was found to comprise a signal peptide, 14 LRR domains in the extracellular region and a TIR domain in the cytoplasmic region, which fits with the characteristic TLR domain architecture. The phylogenetic analysis showed that CcTLR21 possessed high amino acid identities with the TLR21s in other freshwater teleosts. A Real-time PCR assay showed that CcTLR21 mRNA was expressed in almost all tissues examined in healthy common carp, while the levels obviously varied among different tissues. During the embryonic and early larval developmental stages of common carp, the CcTLR21 showed two peaks of expression, with the first at 1 dpf and the second at 10 dpf. When challenged with poly(I:C) (a viral model) or *Aeromonas hydrophila*, the expression level of CcTLR21 was up-regulated in a variety of common carp tissues.

**Conclusions:**

Our findings indicate that CcTLR21 plays a significant role in innate immune defense during larvae ontogeny and in responses to viral or bacterial pathogens.

**Electronic supplementary material:**

The online version of this article (10.1186/s12917-018-1474-4) contains supplementary material, which is available to authorized users.

## Background

Toll-like receptors (TLRs) are one class of pattern recognition receptors (PRRs), which recognize various pathogens-associated molecular patterns (PAMPs) in the host innate immune system, such as lipopolysaccharide (LPS), peptidoglycan, lipoteichoic acid, non-methylated CpG DNA and so on [1, 2]. To date, 11 kinds of TLRs have been identified in humans, 13 in mice and 17 in fish [3, 4]. Among these, TLR21 is a kind of non-mammalian TLR found in fish, birds and amphibians; some examples are chicken (*Gallus gallus*) [5], south African clawed frog (*Xenopus laevis*) [6], zebrafish (*Danio rerio*) [7], grass carp (*Ctenopharyngodon idella*) [8], orange-spotted grouper (*Epinephelus coioides*) [9], channel catfish (*Ictalurus punctatus*) [10], Atlantic salmon (*Salmo salar*) [11], turbot (*Scophthalmus maximus*) [12], large yellow croaker (*Larimichthys crocea*) [13], yellow catfish (*Pelteobagrus fulvidraco*) [14], rock bream (*Oplegnathus fasciatus*) [15] and yellowtail (*Seriola lalandi*) [16].

TLRs are type-I transmembrane proteins that are composed of three domains: an intracellular Toll/interleukin-1 receptor (TIR) domain, a transmembrane region and an extracellular leucine-rich repeat (LRR) domain. The LRR domain binds to PAMPs, and the TIR domain transmits signals into the cytosol by recruiting Myeloid differentiation factor 88 (MyD88) or TIR-domain-containing adapter-inducing interferon-β (TIRF) [17], which are responsible for the production of pro-inflammatory cytokines or type I interferons [18]. Previous studies reported that fish TLR21 could recognize non-methylated CpG DNA and were considered a functional homologue of mammalian TLR9 [12]. Accordingly, upon stimulation with viral stimulants, such as turbot reddish body iridovirus (TRBIV), infectious salmon anaemia virus (ISAV), rock bream iridovirus (RBIV), polyinosinic: polycytidylic acid [poly(I:C)] or CpG oligodeoxynucleotides (CpG-ODN), the TLR21 mRNA expression was up-regulated in turbot [12], Atlantic salmon [11], large yellow croaker [13], yellow catfish [14] and rock bream [15]. In addition, upon stimulation with bacteria, such as *Aeromonas hydrophila*, *Streptococcus iniae*, *Edwardsiella tarda*, *Vibrio alginolyticus* or *Vibrio parahaemolyticus,* the mRNA expression levels of the TLR21 gene were also up-regulated in grass carp [8], large yellow croaker [13], yellow catfish [14] and rock bream [15]. This suggests that fish TLR21s play key roles in immune defense against both viral and bacterial infections.

Common carp (*Cyprinus carpio* L.) is a freshwater fish widespread in Europe and Asia. To date, TLR1 and TLR2 [19, 20], TLR3 [21], TLR4 [22], TLR9 [23], TLR18 [24], TLR20 [25] and TLR22 [26] were reported in common carp. Since TLRs play an important role in host anti-pathogen responses, the study of TLRs will be beneficial to the disease control of common carp. In this study, we reported the cloning and identification of a TLR21 cDNA from common carp (named CcTLR21). We analysed the gene expression profiles of CcTLR21 in various tissues and during embryonic and early larval developmental stages of common carp. Moreover, the gene expression of CcTLR21 is studied after viral or bacterial stimulation to speculate on the possible role of TLR21 in fish immune response against pathogenic infections.

## Methods

### Fish rearing and sample collection

Common carp, with an average weight of 75 g, were obtained from the Fresh Water Fishery Research Institute of Shandong Province. Before the start of the experiment, the fish were reared at 20–25 °C in a recirculating freshwater system for at least two weeks, and fed once a day with commercial carp diet. The fish were euthanatized by immersion in a solution of Tricaine Methane Sulfonate (MS222, Sigma Aldrich) at a concentration of 100 mg/l of water, and the tissue samples obtained from three healthy common carp, including liver, spleen, head kidney, foregut, hindgut, gills, skin, brain, gonad, muscle and buccal epithelium, were separately frozen in liquid nitrogen until use for RNA extraction.

To study the gene expression of CcTLR21 during the embryonic and early larval stages, four pairs of parent fish were selected for artificial propagation. Fertilized eggs were incubated in water reservoir at 28–30 °C with enough oxygen. After fertilization, the embryonic stage of common carp is from one to two days, and the hatching was at three days post fertilization (dpf). At 1, 2, 3, 4, 6, 10, 16 and 24 dpf, embryo or larvae samples were sampled randomly for RNA extraction (three repeats for each time piont).

### Viral and bacterial challenges in vivo

The fish for the in vivo challenge were divided equally into two independent groups. One group was injected intraperitoneally with formalin-inactivated *A. hydrophila* with 5 × 10^7^ CFU per fish [27–29], and the other group injected intraperitoneally with 500 μl of poly(I:C) (SIGMA) solution per fish at a dose of 1.6 mg/ml [30, 31]. After challenge, the tissues (liver, spleen, head kidney, foregut and hindgut) of fish were sampled at different time points from three fish in each group, and total RNA were extracted (Tiangen) and reverse transcripted to cDNA (Tiangen).

### Cloning and analysis of CcTLR21 cDNA

Primers TLR21 F1 and TLR21 R1, which were designed based on the conserved regions of the other species TLR21 sequences, were used to amplify the cDNA fragment of CcTLR21 from the head kidney of common carp. PCR was performed with the following setting: 30 cycles of 94 °C for 30 s, 55 °C for 45 s, and 72 °C for 1 min. The PCR products were ligated into the pMD18-T vector and transformated into competent *E. coli* DH-5α for sequencing. The full-length of the TLR21 cDNA were obtained by RACE (rapid amplification of the cDNA ends) using the 3′-full and 5′-full RACE core set (TaKaRa). The primers used are shown in Table 1.

**Table 1:** The primers and their applications in this study

Primer	Sequence (5` → 3`)	Application
TLR21 F1 TLR21 R1	CTACAGTTTCAGGAGTTGCA TACGATTGTATCGATAGCTCAG	cDNA amplification
5′- GSP Outer 5′- GSP Inner 3′- GSP Outer 3′- GSP Inner	TGGCAAGCGAGTTTGGTAGACGGGTGGA TAGACGGGTGGAGTGATGCAGGGTG CCAGCTATCGTCTCTTTCAAGA GCAACYGTCTACTTATCACC	RACE gene specific primers
TLR21 F2 TLR21 R2 40S F 40S R	AAGGACCAGGAGGAGAAAT AGAGCCGAAATGAAGAACC CCGTGGGTGACATCGTTACA TCAGGACATTGAACCTCACTGTCT	Real-time PCR

The structural domains of CcTLR21 were characterized using the SMART (a simple modular architecture research tool) program (http://smart.embl-heidelberg.de/). The amino acid sequence alignment of TLR21s was performed with MegAlign in DNAstar 7.0 using the method of Clustal W. The phylogenetic tree was generated with MEGA 6.0 using the Neighbour-Joining method.

### Real-time PCR

The Real-time PCR analysis of CcTLR21 gene expression was performed with a Rotor-Gene Q PCR instrument (Qiagen) using SYBR Green Real Master Mix (Tiangen). The amplification scheme was: incubated for 1 min at 94 °C, followed by 40 cycles of 20 s at 94 °C, 20 s at 59 °C and 50 s at 70 °C. For each mRNA, gene expression was corrected by the 40S ribosomal protein S11 in each sample. Relative expression of CcTLR21 mRNA was determined using the 2^(-ΔΔCt)^ method. The primers used are shown in Table 1. In all cases, each PCR was performed with triplicate samples.

### Statistical analysis

Differences in relative gene expression between the challenged group and the control group were analysed using the Graphpad Prism 6 and were considered significant when *p* < 0.05. A two-way analysis of variance (ANOVA) was performed to test differences in gene expression in each tissue.

## Results

### cDNA sequence of CcTLR21

**Fig. 1:**
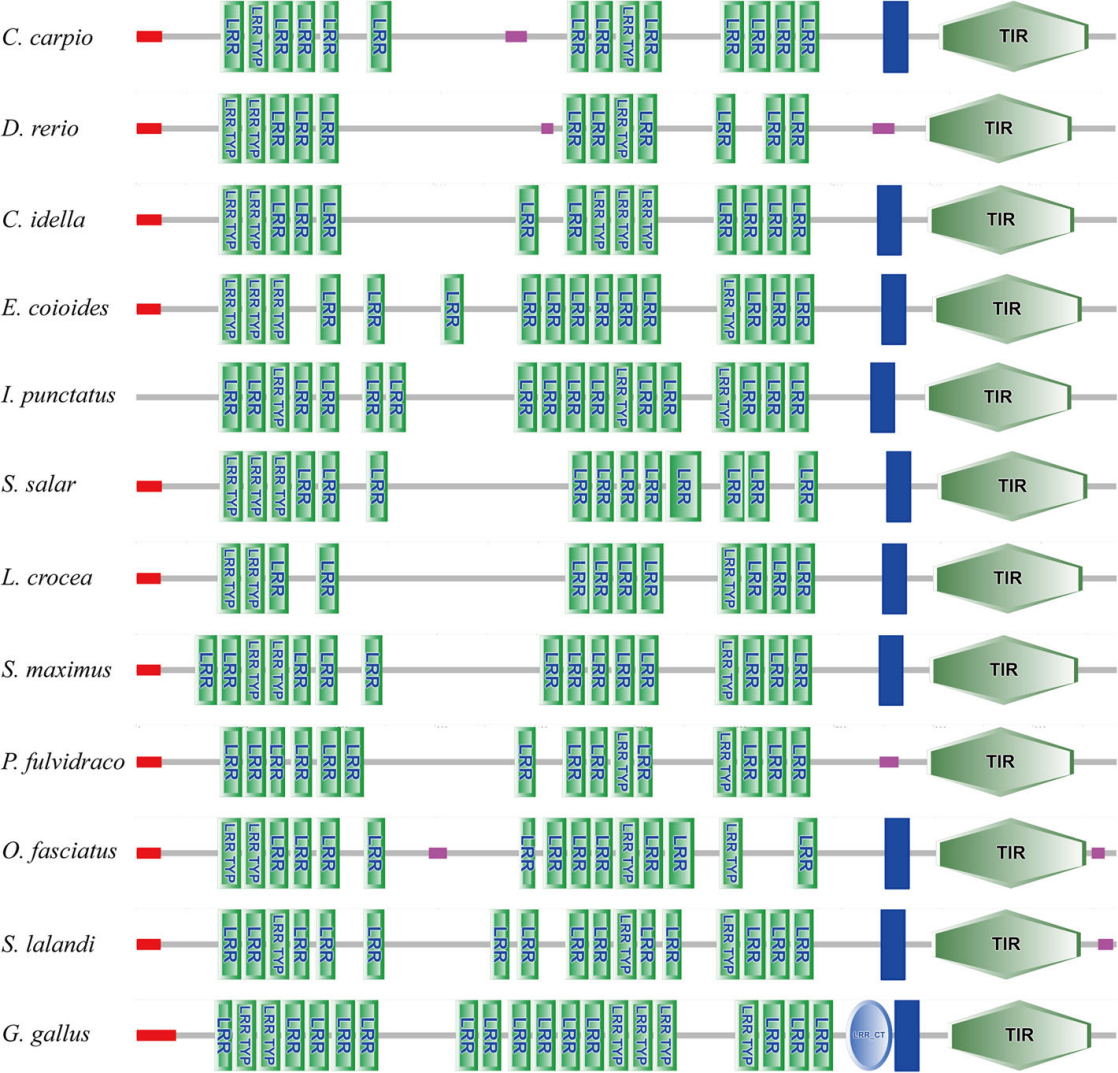
The domain organizations for TLR21 in various species. Schematic representation of TLR21 domains was predicted by SMART. N-terminal signal peptide (red box), LRRs and LRR-CT domain are denoted at the extracellular region, followed by a transmembrane domain (blue box), and a TIR domain in the cytoplasmic region

The full-length CcTLR21 cDNA (GenBank accession number MF615210) was amplified from the head kidney of common carp, which was 3557 bp long, including a 149 bp 5`-untranslated region (UTR), an open reading frame (ORF) of 2895 bp and a 513 bp 3`-UTR. The ORF of CcTLR21 encoded a putative protein of 964 amino acids. Using the SMART program, the CcTLR21 protein was found to comprise a signal peptide (24 amino acids), 14 LRR domains in the extracellular region and a TIR domain in the cytoplasmic region (Fig. 1).

### Homology alignment and phylogenetic analysis of TLR21

**Fig. 2:**
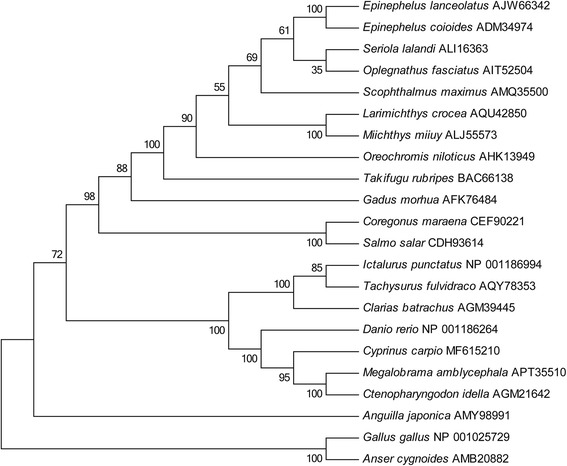
Phylogenetic analysis of TLR21 amino acid sequences. The evolutionary tree of known TLR21s in fish and birds. These trees are generated using the method of Neighbour-Joining in MEGA 6. The GenBank accession numbers of these sequences are shown in the trees

Homology alignment analysis showed that the deduced CcTLR21 protein exhibited significant similarity (from 43.4% to 81.7%) to other known TLR21s (Additional file 1: Table S1). Moreover, the amino acid sequences of CcTLR21 showed a relatively higher degree of similarity with other fish TLR21 than with bird TLR21. The TIR domain of TLR21 presented three conserved regions: box 1 (YDXFXSY), box 2 (LCLHHRDFXPG) and box 3 (FWXXLXXA), which were all found through multiple sequence alignment with the TLR21 family proteins (Additional file 2: Figure S1). To investigate the evolutionary relationships of TLR21 in fish and birds, a phylogenetic tree was constructed. In the tree, all teleost TLR21 members were separated from the TLR21 in birds (Fig. 2).

### Constitutive expression of the CcTLR21 gene in common carp

**Fig. 3:**
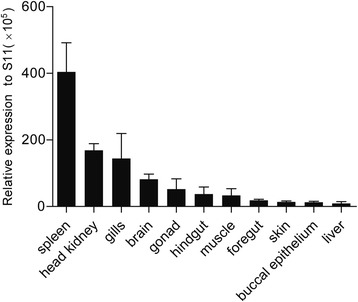
Tissue expression of CcTLR21 in healthy common carp. CcTLR21 transcripts in the spleen, head kidney, gills, brain, gonad, hindgut, muscle, foregut, skin, buccal epithelial and liver of common carp are detected by Real-time PCR. Amplification of 40S ribosomal protein S11 in each tissue is performed as an internal control. *n* = 3

The expression of CcTLR21 mRNA was found in almost all tissues examined, but the levels varied strongly between different tissues. The highest expression level of CcTLR21 mRNA was detected in the spleen, head kidney and gills, with a moderate level of expression observed in the brain, gonad, hindgut and muscle, while very low expression was observed in the foregut, skin, buccal epithelium and liver (Fig. 3).

**Fig. 4:**
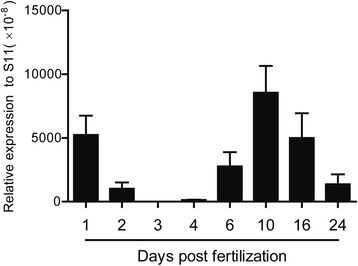
CcTLR21 gene expression during common carp larvae ontogeny. A normalized expression of CcTLR21 gene during the development of common carp larvae between 1 and 24 days post fertilization. Amplification of 40S ribosomal protein S11 in each tissue is performed as an internal control. *n* = 3

We analyzed the constitutive expression of the CcTLR21 gene in embryo and early larvae of common carp from 1 to 24 days post fertilization (dpf). The results showed that CcTLR21 have two peaks of expression, with the first at 1 dpf and the second at 10 dpf (Fig. 4).

### Expression profiles of CcTLR21 gene in common carp upon poly(I:C) challenge

**Fig. 5:**
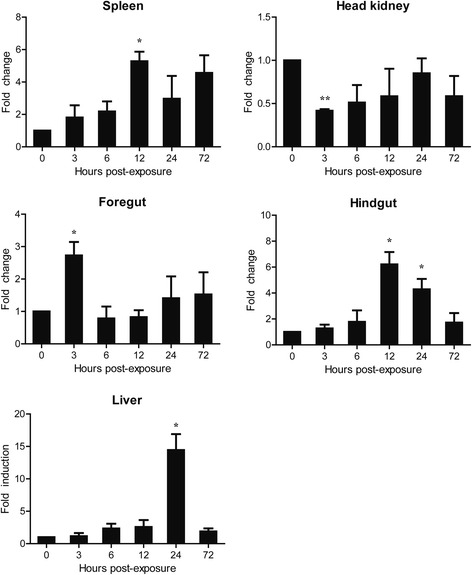
The relative expression of CcTLR21 in common carp after i.p. injection with poly(I:C). The relative expression of CcTLR21 in the spleen, head kidney, foregut, hindgut and liver of common carp was examined at different time points by Real-time PCR. All these results are corrected by 40S ribosomal protein S11. Data are presented as a fold increase of the challenged group to the un-stimulated control group (denoted by 0 h) and shown as the mean ± SEM (*n* = 3). *p < 0.05 or **p < 0.01 versus un-stimulated fish

The expression level of CcTLR21 mRNA in common carp after poly(I:C) stimulation was up-regulated in the spleen, foregut, hindgut and liver (Fig. 5, *p* < 0.05 or *p* < 0.01), with an increase of 5.29-, 2.73-, 6.22-, and 14.44-fold, respectively. The highest induced expression level was found at 3 hpi in the foregut, 12 hpi in the spleen and hindgut, and 24 hpi in the liver. However, CcTLR21 expression was down-regulated in the head kidney at 3 hpi (*p* < 0.01).

### Expression profiles of CcTLR21 gene in common carp upon *A. hydrophila* challenge

**Fig. 6:**
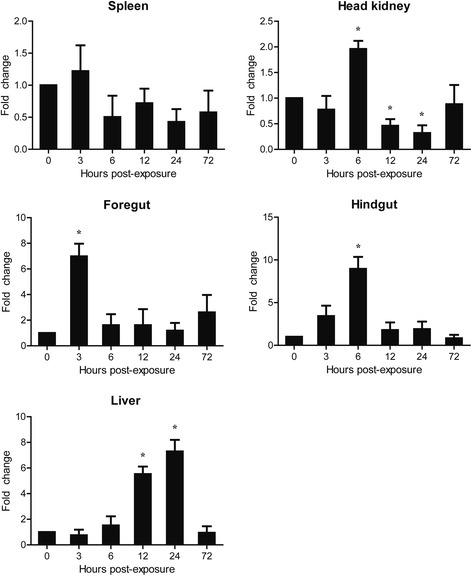
The relative expression of CcTLR21 in common carp after i.p. injection with *A. hydrophila*. The relative expression of CcTLR21 in the spleen, head kidney, foregut, hindgut and liver of common carp was examined at different time points by Real-time PCR. All these results are corrected by 40S ribosomal protein S11. Data are presented as a fold increase of the challenged group to the un-stimulated control group (denoted by 0 h) and shown as the mean ± SEM (*n* = 3). *p < 0.05 or **p < 0.01 versus un-stimulated fish

After i.p. injection with formalin-inactivated *A. hydrophila*, the expression level of CcTLR21 mRNA was up-regulated in the head kidney, foregut, hindgut and liver of common carp (Fig. 6, *p* < 0.05), with an increase of 1.96-, 6.99-, 8.95-, and 7.30-fold, respectively. The highest induced expression level was found at 3 hpi in the foregut, 6 hpi in the head kidney and hindgut, and 24 hpi in the liver. However, CcTLR21 expression remained unchanged in the spleen after *A. hydrophila* challenge.

## Discussion

In the present study, the full-length CcTLR21 cDNA was first amplified from common carp. The ORF of CcTLR21 encoded a putative protein of 964 amino acids, which was found to comprise a signal peptide, 14 LRR domains in the extracellular region and a TIR domain in the cytoplasmic region. This represents the characteristic TLR domain architecture, although the number of LRR domains in different fish TLR21s differs greatly. Like CcTLR21, the TLR21 of *C. idella* and *S. salar* exhibit 14 LRR domains, while the TLR21 of *D. rerio* and *L. crocea* contain 12, *P. fulvidraco* and *O. fasciatus* contain 15, *E. coioides* and *S. maximus* contain 16, *S. lalandi* contains 17, *I. punctatus* contains 18 and *G. gallus* contains 21 LRR domains (Fig. 1). The LRR motif was involved in ligand recognitions that bind to PAMPs for the purpose of subsequent signal transduction [32, 33]. The exact reasons for species-wise variations in number of LRR remain unknown, but the variation in the number of LRR domains suggest that fish TLR21 may exhibit a special mode of ligand binding [34, 35].

The TIR domain of CcTLR21 presented three conserved regions: box 1 (YDXFXSY), box 2 (LCLHHRDFXPG) and box 3 (FWXXLXXA), which were also found in other species TLR21 proteins (Additional file 2: Figure S1). The conserved box 1 and box 2 motifs were known to mediate the coupling of receptor molecules in signal transduction pathways, while the function of box3 region was to control subcellular location of these receptors [12]. Thus, the TIR domain is structurally and functionally conserved among different species and seems to trigger similar intracellular signal transduction pathways.

To investigate the evolutionary relationships of TLR21 in fish and birds, a phylogenetic tree was constructed in which all teleost TLR21 members were separated from the TLR21 in birds. As for teleost TLR21, there were two distinct subgroups, one of which consisted of marine teleost TLR21 proteins, including TLR21 of *S. salar, C. maraena, E. coioides, E. lanceolatus, S. lalandi, O. fasciatus, T. rubripes, S. maximus, O. niloticus, M. miiuy, L. crocea* and *G. morhua*; the other was comprised of freshwater teleost TLR21 proteins, including *C. carpio, M. amblycephala, C. idella, D. rerio, I. punctatus, C. batrachus* and *T. fulvidraco*. Interestingly, the TLR21 of *A. japonica* was in a signal branch. The result suggested that TLR21 in marine and freshwater teleosts might undergo different evolutionary processes.

The expression of CcTLR21 mRNA was found in almost all examined tissues of the healthy common carp, but the levels varied strongly among different tissues. In fish, the spleen and head kidney are important systemic lymphoid organs [36], and a high expression of CcTLR21 in these organs suggests an important role of CcTLR21 in the immunity of common carp. Similar high expression of the TLR21 gene in the spleen and head kidney was found in grass carp [8], orange-spotted grouper [9], Atlantic salmon [11], large yellow croaker [13], yellow catfish [14], rock bream [15] and yellowtail [16]. Meanwhile, the gills, hindgut, foregut, buccal epithelium and skin are mucosal immune organs in fish. The high expression level of the CcTLR21 gene in the gills and hindgut suggests that CcTLR21 might play a role in the mucosal defenses of common carp, although there is much lower expression in the foregut, skin and buccal epithelium. Similarly, high expression of the TLR21 gene in gills was found in grass carp [8], orange-spotted grouper [9], Atlantic salmon [11], large yellow croaker [13] and rock bream [15]. In addition, in grass carp [8], large yellow croaker [13] and yellowtail [16], the TLR21 gene also had high expression in the intestine and skin, indicating that the tissue expression pattern of TLR21 varied among different fish species.

We analyzed the constitutive expression of the CcTLR21 gene in embryo and early larvae of common carp post fertilization. The results showed that CcTLR21 had two peaks of expression. Similarly, in yellow catfish, the expression of TLR21 mRNA was at a high level from the fertilized egg stage to the late blastula stage, and subsequently showed an significant up-regulation from 1 to 30 days post hatching (dph) [14]. Additionally, the mRNA expression of other TLRs during embryonic development of fish, such as TLR3, TLR5, TLR18 and TLR19 genes were also reported. The TLR3 mRNA had a higher expression at the fertilized egg stage than at other embryonic developmental stages in rohu [37], and the expression level of TLR5 mRNA was the highest at 5 h post fertilization in mrigal [38]. In yellow catfish, the expressions of TLR18 and TLR19 mRNA were high during the early-stage embryonic development from the fertilized egg to the late blastula [14]. These results suggested that the mRNAs of maternal TLRs might be transferred to the fertilized eggs of fish and will be gradually consumed during the embryonic developmental stage [39]. After hatching, the CcTLR21 mRNA expression levels increased in common carp. In line with this, the mRNA expressions of other TLR genes have also been reported to be up-regulated post hatching of fish, such as rohu, mrigal and yellow catfish [14, 37, 38]. All these results suggest that TLRs may be involved in some immune-related activities, as the larvae will be exposed to the complicated water environment after hatching. Therefore, CcTLR21 might play important immune roles in the embryonic and early larval developmental stages of common carp.

In some reported fish species, significant up-regulation of the TLR21 mRNA was observed after viral or bacterial stimulation, suggesting a possible immune function of fish TLR21. Accordingly, in the present study, we analysed the expression pattern of CcTLR21 mRNA after poly(I:C) and *A. hydrophila* challenges in a variety of immune-related tissues.

Poly(I:C) is used here as a model of double-stranded genome virus infection [40, 41]. After poly(I:C) challenge, the expression level of CcTLR21 mRNA was up-regulated in the spleen, foregut, hindgut and liver. Similarly, upon stimulation with poly(I:C), TRBIV and CpG-ODN, the TLR21 mRNA expression was up-regulated in the gills, head kidney, spleen and muscle of turbot [12]. Additionally, three CpG-ODNs were found to significantly up-regulate the expression of TLR21 in large yellow croaker head kidney cells [13], and TLR21 mRNA levels significantly increased in the spleen tissues of rock bream in response to RBIV infection [15]. The changes of gene expression induced by various infections might be due to the cell migration or proliferation, or actual modulation of gene transcription in resident cells. Although the viral stimulation and subsequent up-regulation of TLR21 gene expression does not claim the recognition of specific ligands by TLR21, induction of TLR21 gene expression is consistent over different studies across different fish species, at least indicating the involvement of fish TLR21 in innate immune defense against viral pathogens [42]. Otherwise, in the head kidney of common carp, the CcTLR21 expression was down-regulated upon poly(I:C). Similarly, the TLR21 expression was down-regulated in the kidneys of Atlantic salmon after ISAV infection [11], and in the liver and spleen of grass carp upon aquareovirus induction [8]. The down-regulation of TLR21 gene expression indicates an altered rate of transcription or a migration of the relevant cell type away from the tissues, implying that TLR21 may have different roles in different tissues of fish.

In the present study, *A. hydrophila,* a Gram-negative bacterium found in fresh or brackish water and associated with some diseases of freshwater fish and amphibians [24, 26, 29, 31], was performed to investigate the possible role of CcTLR21 in the immune defenses against bacterial pathogens in common carp. After i.p. injection with formalin-inactivated *A. hydrophila*, the expression level of CcTLR21 mRNA was up-regulated in the head kidney, foregut, hindgut and liver of common carp. Similar results have been reported in some other fish species. After challenge with *A. hydrophila*, the expression level of the TLR21 gene was up-regulated in the spleen, head kidney, trunk kidney, liver and blood of yellow catfish [14], and in the liver and spleen of grass carp [8]. Post *C. irritans* infection, TLR21 transcript was induced in the skin and gill of orange-spotted grouper [9]. Upon stimulation with *S. iniae* or *E. tarda*, TLR21 mRNA levels was significantly up-regulated in the spleen of rock bream [15]. The expression of TLR21 gene was quickly increased in the spleen and head kidney of large yellow croaker, in response to a trivalent bacterial vaccine consisting of *V. alginolyticus*, *V. parahaemolyticus*, and *A. hydrophila* [13]. Thus, similar to the results of viral stimulation, the induced expression of TLR21 indicates its potential role in the innate immune response of fish against bacterial pathogens.

## Conclusions

The structure, evolutionary relationship and expression characteristics of a TLR21 gene in common carp were reported in the present study. CcTLR21 seems to have a closer evolutionary relationship with other freshwater fish TLR21s than those of marine fish species. The constitutive expression of CcTLR21 in various tissues and during early larval ontogeny implies its possible relevance to immune function of common carp. Moreover, the up-regulated expression of CcTLR21 strongly indicates that CcTLR21 plays a significant role in innate immune defense against viral and bacterial pathogenic microbes.

## Additional files

Additional file 1: **Table S1.** Percent identity of CcTLR21 with other species. (DOCX 15 kb)

Additional file 2: **Figure S1.** Alignment of CcTLR21 with other species TLR21s. The signal peptide, LRRs domain, transmembrane region and TIR domain were denoted, respectively. The three active motifs in TIR domain are boxed: box 1 (YDXFXSYN), box 2 (LCLHHRDFXXG) and box 3 (FWXXL). X denotes an arbitrary amino acid. (TIF 3641 kb)

## Abbreviations

ANOVA: two-way analysis of variance
CpG-ODN: CpG oligodeoxynucleotides
dpf: days post fertilization
dph: days post hatching
ISAV: infectious salmon anaemia virus
LB: Luriae Bertani
LPS: lipopolysaccharide
LRR: leucine-rich repeat
MyD88: Myeloid differentiation factor 88
ORF: open reading frame
PAMPs: pathogen-associated molecular patterns
poly(I:C): polyinosinic: polycytidylic acid
PRRs: pattern recognition receptors
RACE: rapid amplification of the cDNA ends
RBIV: rock bream iridovirus
SMART: a simple modular architecture research tool
TIR: Toll/interleukin-1 receptor
TIRF: TIR-domain-containing adapter-inducing interferon-β
TLR: toll-like receptor
TRBIV: turbot reddish body iridovirus
UTR: untranslated region
