# Supplementary material for: Molecular characterization and expression patterns of a non-mammalian toll-like receptor gene (TLR21) in larvae ontogeny of common carp (Cyprinus carpio L.) and upon immune stimulation
Source: BMC Vet Res. 2018 May 3;14:153. doi: 10.1186/s12917-018-1474-4 (PMC5934810; doi:10.1186/s12917-018-1474-4)
Supplement: Supplementary file 1 — Table S1. Percent identity of CcTLR21 with other species. (DOCX 15 kb) [file 12917_2018_1474_MOESM1_ESM.docx]

**Table S1. Percent identity of CcTLR21 with other species.**

| **species** | **percent identity** |
| --- | --- |
| *Cyprinus carpio*  *Megalobrama amblycephala*  *Ctenopharyngodon idella*  *Danio rerio*  *Ictalurus punctatus*  *Clarias batrachus*  *Tachysurus fulvidraco*  *Salmo salar*  *Coregonus maraena*  *Epinephelus coioides*  *Epinephelus lanceolatus*  *Seriola lalandi*  *Oplegnathus fasciatus*  *Anguilla japonica*  *Takifugu rubripes*  *Scophthalmus maximus*  *Oreochromis niloticus*  *Miichthys miiuy*  *Larimichthys crocea*  *Gadus morhua*  *Gallus gallus*  *Anser cygnoides* | 100  81.7  80.7  76.6  62.1  61.8  60.9  56.5  56.2  55.3  54.7  54.7  54.4  53.9  53.5  53.2  53.2  51.8  51.2  50.5  43.4  43.4 |
