# Supplementary figures and images for: Molecular characterization and expression patterns of a non-mammalian toll-like receptor gene (TLR21) in larvae ontogeny of common carp (Cyprinus carpio L.) and upon immune stimulation
Source: BMC Vet Res. 2018 May 3;14:153. doi: 10.1186/s12917-018-1474-4 (PMC5934810; doi:10.1186/s12917-018-1474-4)

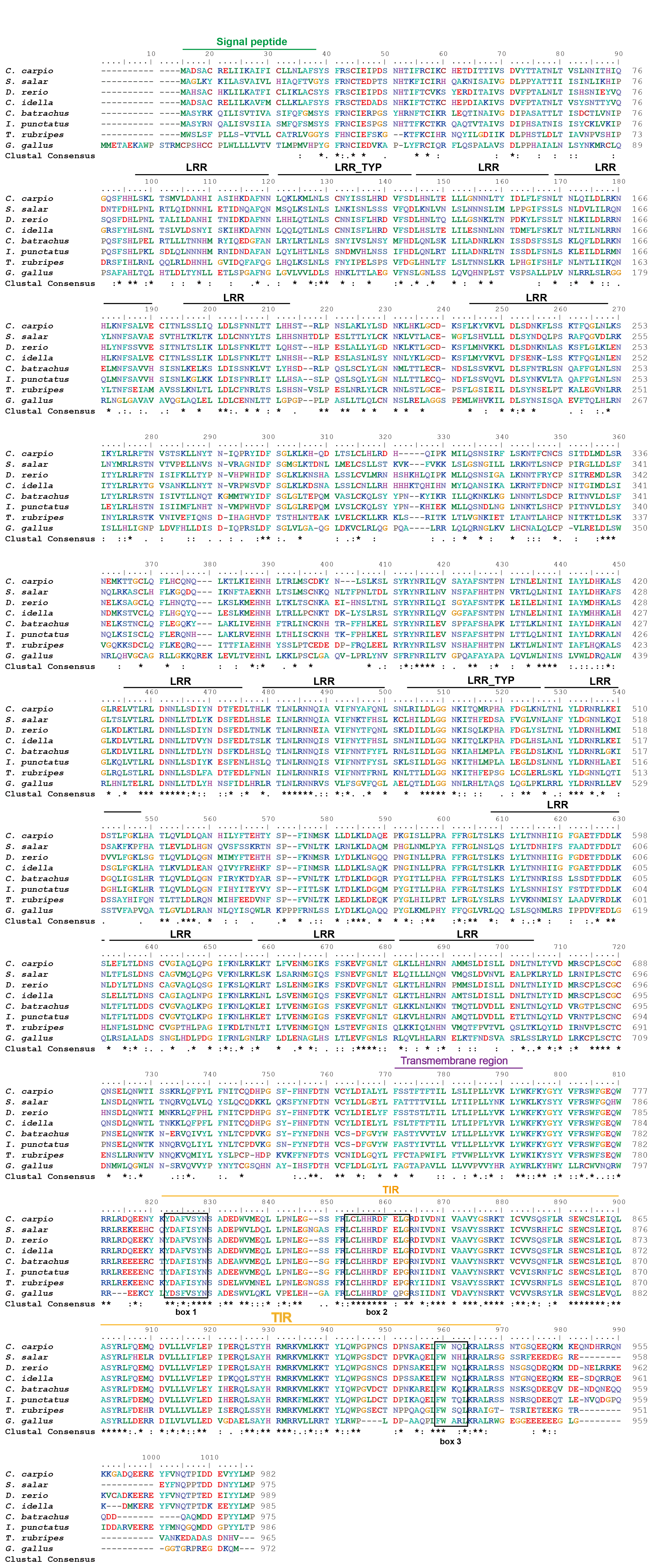

Supplement: Supplementary file 2 — Figure S1. Alignment of CcTLR21 with other species TLR21s. The signal peptide, LRRs domain, transmembrane region and TIR domain were denoted, respectively. The three active motifs in TIR domain are boxed: box 1 (YDXFXSYN), box 2 (LCLHHRDFXXG) and box 3 (FWXXL). X denotes an arbitrary amino acid. (TIF 3641 kb) [file 12917_2018_1474_MOESM2_ESM.tif]
